# Exploration of potential risks of Hand, Foot, and Mouth Disease in Inner Mongolia Autonomous Region, China Using Geographically Weighted Regression Model

**DOI:** 10.1038/s41598-018-35721-9

**Published:** 2018-12-07

**Authors:** Zhimin Hong, Hui Hao, Chunyang Li, Wala Du, Lidong Wei, Huhu Wang

**Affiliations:** 1 School of Sciences, Inner Mongolia University of Technology, Department of Mathematics, Hohhot, 010051 China; 2 The Affiliated Hospital of Inner Mongolia Medical University, Department of Neurology, Hohhot, 010050 China; 3 Ecological and Agricultural Meteorology Center of Inner Mongolia Autonomous Region, Hohhot, 010051 China; 4 Inner Mongolia Hohhot Bureau of Statistics, Department of Research, Hohhot, 010011 China; 5 Inner Mongolia Autonomous Region Center for Disease Control and Prevention, Institute for infectious disease and endemic disease control, Hohhot, 010031 China

## Abstract

To quantify the associations between the spatial characteristics of hand, foot, and mouth disease (HFMD) epidemic and meteorological factors (average temperature (AT), relative humidity (RH), average pressure (AP), average wind speed (AW) and average rainfall (AR)), child population density (CPD) and Per capita GDP (GDP) in Inner Mongolia Autonomous Region, China, and to detect the variation of influence in different seasons and counties, geographically weighted regression (GWR) model was constructed. The monthly cumulative incidence (CI) of HFMD was worked out for children ≤9 years from June to December, 2016. The results revealed that GWR model had a far superior goodness-of-fit for describing the relationship between the risk factors and HFMD incidence. Meteorological factors had different significance in their effect on HFMD incidence depending on the season. AT and AR had the greatest impact on HFMD in summer. The influence of RH on HFMD was significant in early autumn. AW was negatively correlated with HFMD in summer and positively correlated in autumn and winter. The effects of AW and AP on the incidence of HFMD were statistically significant in winter. GDP and CPD were not significantly related to HFMD occurrence for most time periods.

## Introduction

Hand, foot, and mouth disease (HFMD) is an acute infectious disease caused by a set of enteroviruses, and it mainly occurs among children under 5 years^1^, the main clinical symptoms of which include fever, skin rash or herpes on hands, feet, buttocks and vesicles in the mouth. Most HFMD cases have mild symptoms and are self-limited, while severe cases have a high case fatality rate. The enteroviruses that cause HFMD are most common in coxsackievirus A16 (CVA16) and enterovirus 71 (EV71)^2^. Over the past decades, HFMD disease has spread widely and caused the most severe affection in Asian countries^3–5^. In 2008, there was a large-scale outbreak of HFMD disease in Fuyang, Anhui Province, China^6^. Subsequently, the Chinese Ministry of Health made HFMD disease as a class “C” notifiable disease, in May, 2008. The incidence of HFMD was ranked the first among the legal class “C” infectious diseases from 2009 to 2012^7^. According to statistics, in China, reported disease rates of HFMD reached 1.2 cases per 1,000 person-years during the period of 2010–2012, and HFMD was responsible for 350–900 reported deaths among children each year^8^. Many studies on the potential association between meteorological factors and HFMD incidence have been reported^9–11^.

The geographically weighted regression (GWR) model, as a spatial statistics method to model and analyze the heterogeneity of space, was proposed based on local smooth ideas. By contrast with the “fixed” coefficients estimation models, such as time-series models and the ordinary linear regression (OLS) model, the GWR model allowed the relationships between a dependent variable and a group of independent variables to vary over the geographic space. Because of its introduction, the GWR model has become a major and popular tool and is widely used in various disciplines and fields, such as geology, environmental science, ecology, economics and other research fields^12–16^.

Inner Mongolia Autonomous Region consisting of 102 counties is located in the north border area of China, crosses the Northeast, the North China and the Northwest, which has the vast dry region and semi-dry region, with a total area of 1,183,000 square kilometers, accounting for 12.3% of China’s land area. It is adjacent to eight provinces of China and is the third largest province in China. There were different patterns of HFMD incidence in Inner Mongolia with different seasons. For example, There are two peaks, in summer (from June to August) and late autumn (October), early winter (from November to December), respectively, in Inner Mongolia. Although the burden of HFMD in Inner Mongolia Autonomous Region (Inner Mongolia) is not high, compared with the Southern China, the incidence of HFMD should not be underestimated in Inner Mongolia. In May, 2007, an outbreak of HFMD disease occurred in Jungar Banner, Erdos city, Inner Mongolia^17^. In June, 2012, an outbreak of HFMD disease caused by CoxA16 virus occurred in Bayin Mu Ren Su Mu, Alxa League, Inner Mongolia, and laboratory diagnostic of etiological displayed that the positive rate for CoxA16 virus was 100%^18^. From May 2008 to December 2016, an epidemic of HFMD in Inner Mongolia involved 171,405 cases, 1,279 severe cases and 34 deaths. From 2009 to 2016, the average annual HFMD incidence rate for children 9 years and younger (≤9 years) in Inner Mongolia decreased to 41.36 from 137.51 per 100,000 child population (the statistical data was obtained from the Inner Mongolia Autonomous Region Center for Disease Control and Prevention). At present, there are not yet particularly efficient antiviral drugs and preventive measures to protect against and decrease the incidence of HFMD. Therefore, it could be very useful and important to detect the reasons and key risk factors that conduce to HFMD outbreaks for assisting prediction and control. Previous studies about HFMD mainly paid attention to descriptive statistics of epidemiological characteristics^19–22^ and detecting spatio-temporal clusters in Inner Mongolia, China^23,24^, lacking analysis and modeling based on spatial geographic information, which is detrimental to prevention and control of HFMD in Inner Mongolia, and that the above studies did not address the spatial variation patterns of HFMD along with potential risk factors. In the present study, our aim was to detect and quantify the potential effects of meteorological factors, child population density and Per capita GDP on HFMD occurrence in Inner Mongolia using the GWR model at county level and monthly scale.

## Results

### Statistical analysis

**Figure 1:**
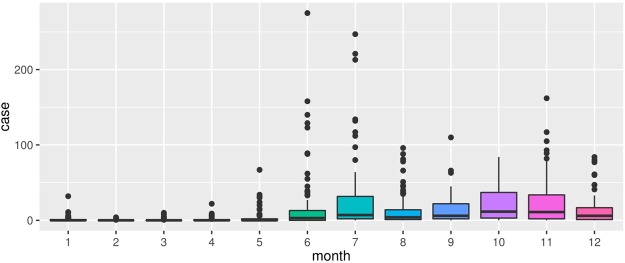
The monthly distribution of HFMD cases in Inner Mongolia, China, 2016.

A total of 13, 928 cases of HFMD were reported to the Inner Mongolia Centre for Disease Control and Prevention surveillance system from 1 January to 31 December, 2016, of which 13, 416 (96.32%) were child cases (aged 0~9 years). The monthly distribution of HFMD cases in Inner Mongolia from 1 January to 31 December, 2016 revealed seasonality in HFMD occurrence (Fig. 1). Summer peaks in the number of HFMD cases were observed in June-August, with secondary peaks occurring in October-December. Therefore, June-December were chosen as the main study period.

### Cumulative Incidence Adjustment and Spatial Autocorrelation Analysis

**Figure 2:**
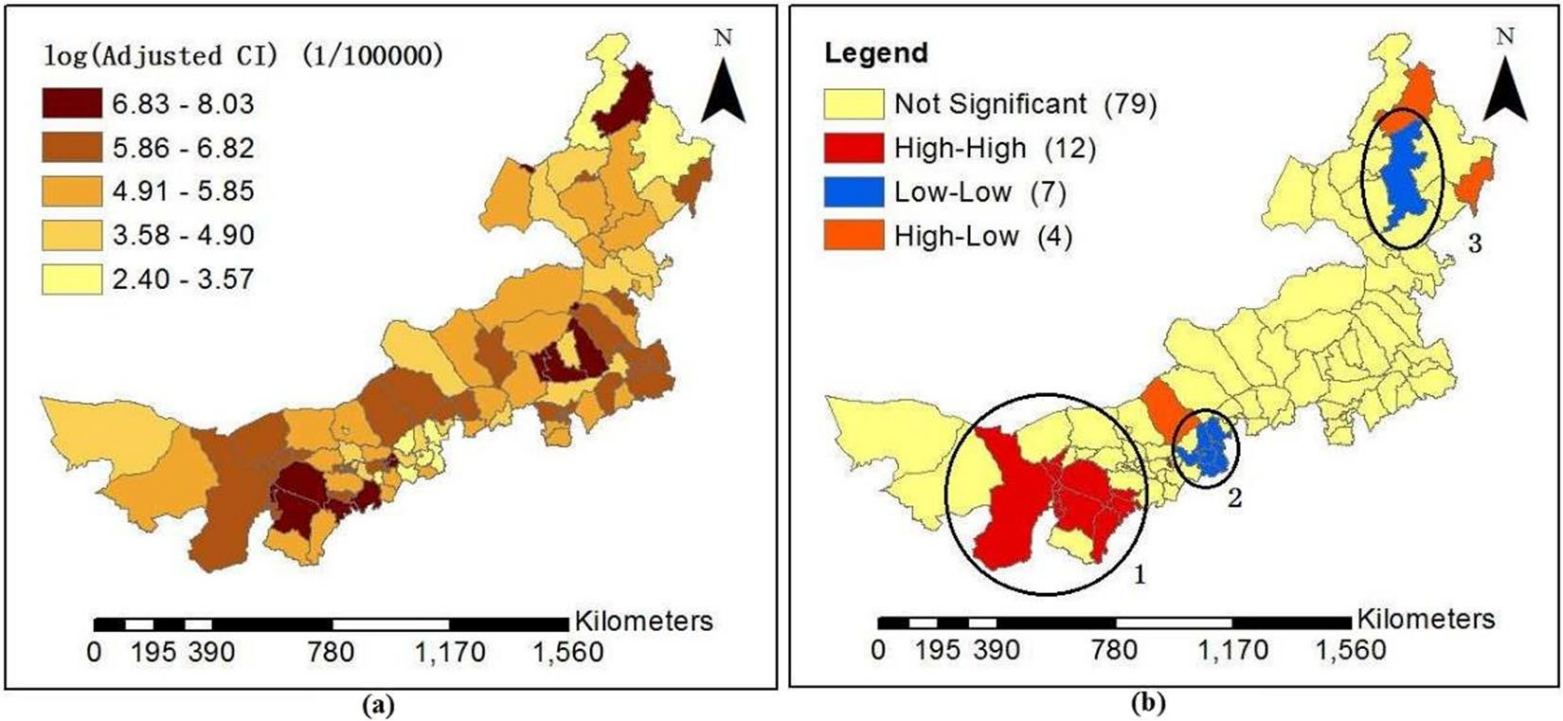
The distribution of the logarithm of adjusted CI of HFMD (**a**) and the Local Moran’s I of the cases of HFMD (**b**) (from June to December, 2016).

12,780 HFMD cases were reported among patients aged 0~9 years in Inner Mongolia from 1 June to 31 December, 2016. The logarithm of adjusted cumulative incidence (CI) (the expected CI of HFMD) is shown in Fig. 2(a). The result indicates that HFMD incidence is higher in mid-east, southwest and northeast counties of Inner Mongolia than other regions. The global Moran’s I index of the adjusted CI is 0.32 (*p* < 0.01), which shows a spatial positive correlation. Figure 2(b) displays the local clusters of the incidence of HFMD by using the local Moran’s I indices, and shows that the high-high cluster regions were located in the southwest of Inner Mongolia, consisting of Dongsheng District, Haibowan District, Ejin Horo Banner, Huimin District, Yuquan District, etc (region 1), and the low-low cluster regions consisted of Shangdu County, Fengzhen District, Zhuozi County, Yakeshi, etc (region 2 and region 3).

### Correlation Analysis on Influencing Factors

Pearson correlation test was performed to explore the potential influence factors for HFMD occurrence (Table 1). The result reveals that the correlation between altitude (ALT) and average pressure (AP) is −0.979. The correlation coefficients are lower among the other variables than the above mentioned. Therefore, the following regression analysis should choose all potential risk factors except altitude (ALT) as explanation variables. The potential risk factors were not statistically significant (*p* ≤ 0.05) based on a global view.

**Table 1:** Correlation analysis and test results.

Factors	CC	Sig.	AT	RH	AP	AW	AR	ALT	CPD	GDP
AT	−0.032	0.392	1							
RH	0.012	0.746	−0.114	1						
AP	0.054	0.150	−0.136	0.234	1					
AW	0.019	0.614	−0.018	−0.341	−0.177	1				
AR	0.006	0.880	0.621	0.355	−0.013	−0.164	1			
ALT	−0.063	0.093	0.041	−0.251	−0.979	0.184	−0.072	1		
CPD	0.040	0.288	0.033	−0.021	−0.075	−0.033	0.025	0.052	1	
GDP	0.062	0.097	0.006	−0.123	−0.234	0.209	−0.067	0.242	−0.016	1

(Notation ‘CC’ represents Correlation Coefficient).

### The seasonal effect of weather factors on HFMD occurrence

**Figure 3:**
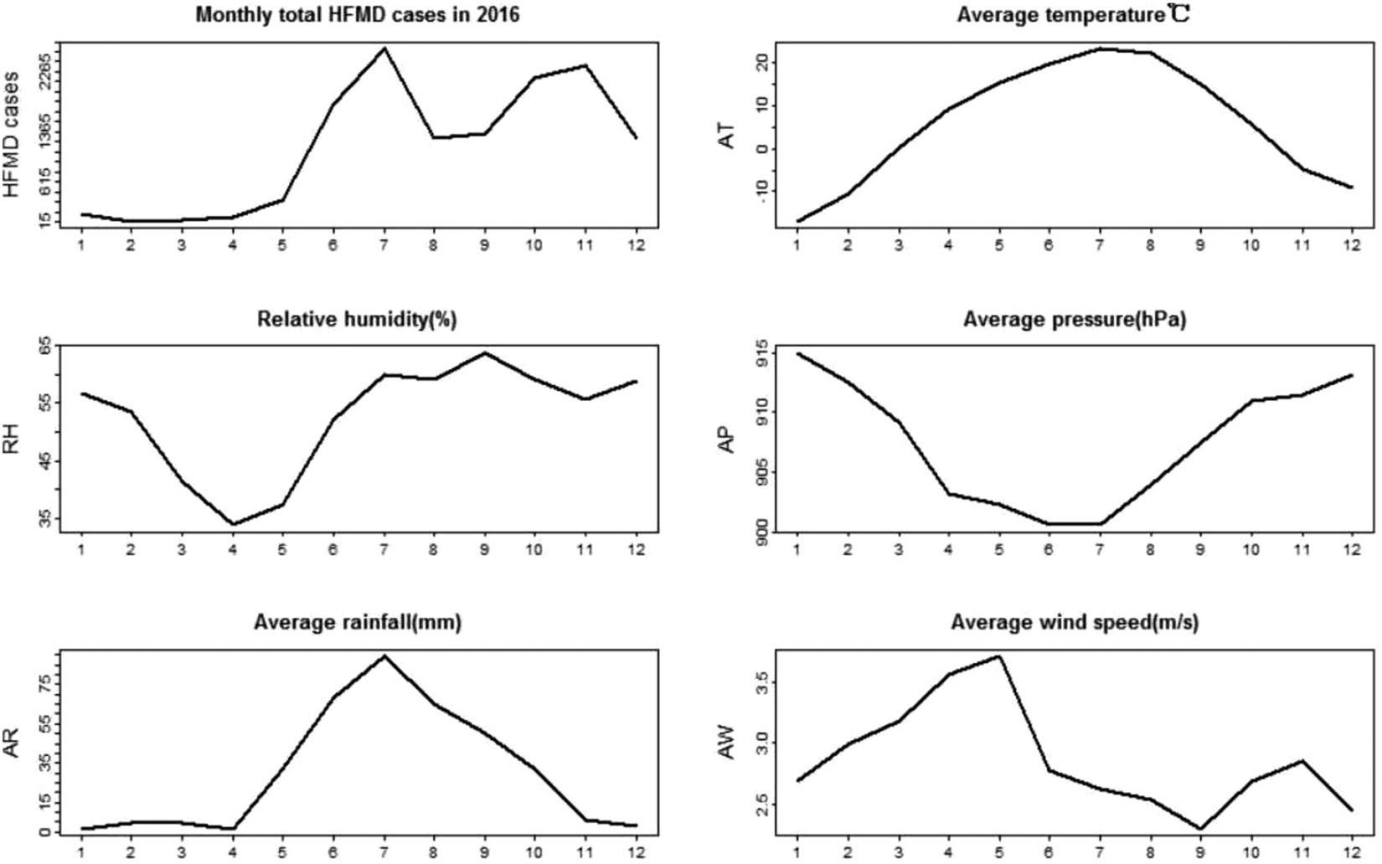
Monthly distribution of HFMD cases and weather factors.

Figure 3 shows the monthly weather factors from January to December, 2016. Average temperature (AT) displays a normal trend. Relative humidity (RH) in Inner Mongolia is lower in spring (from March to May) than other seasons. Average pressure (AP) in Inner Mongolia is lower in summer than other seasons. Average rainfall (AR) predominantly proceeds in June, July and August. Average wind speed (AW) has two peaks in Inner Mongolia, in spring and early winter. Moreover, AW is the lowest in September, 2016.

**Figure 4:**
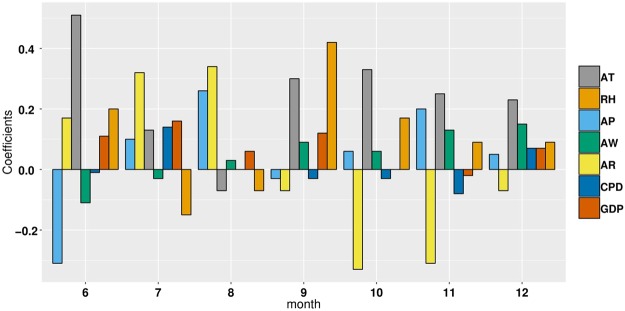
Average coefficients of risk factors.

**Figure 5:**
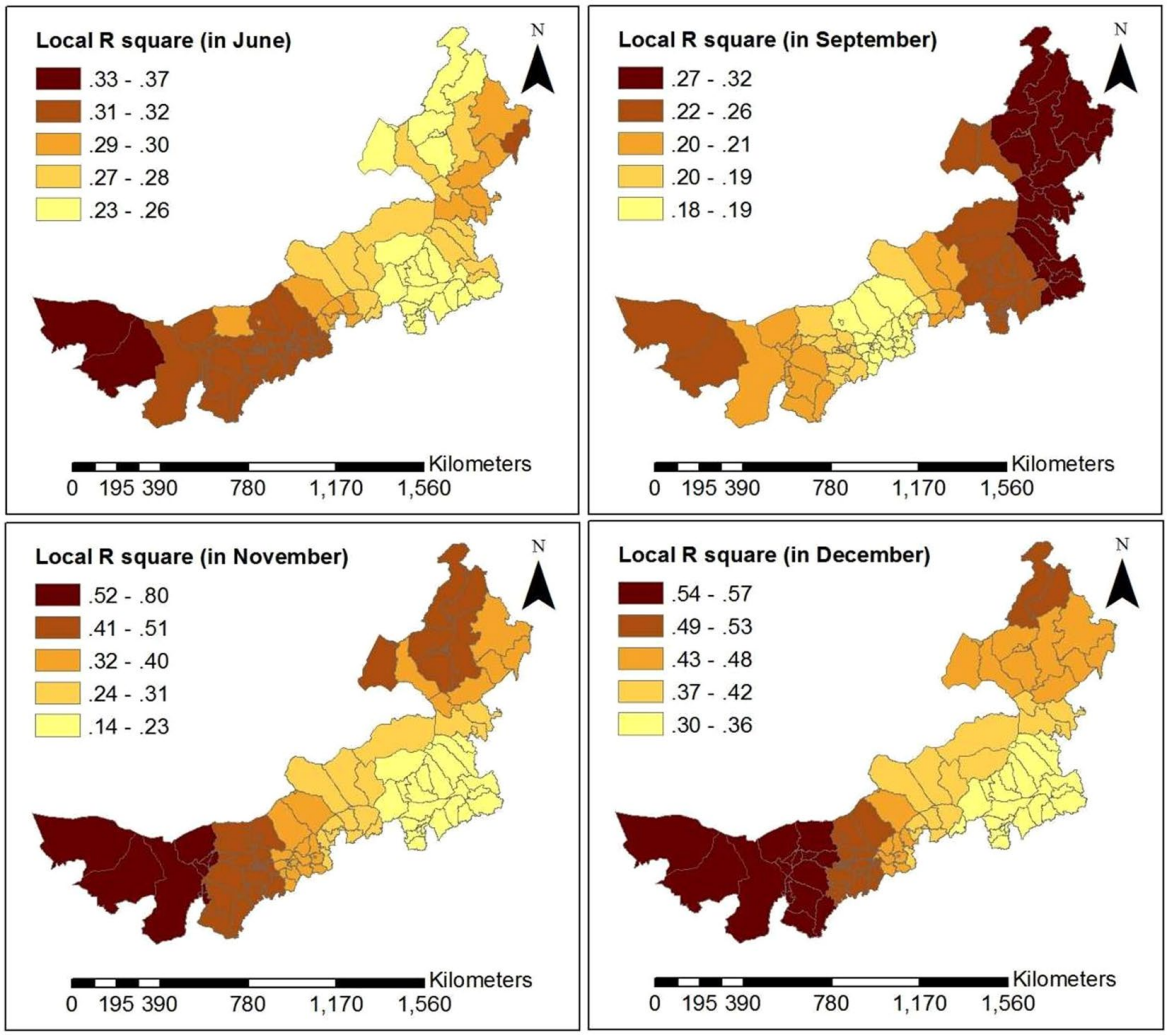
Map of the local R square values obtained from the GWR model.

**Figure 6:**
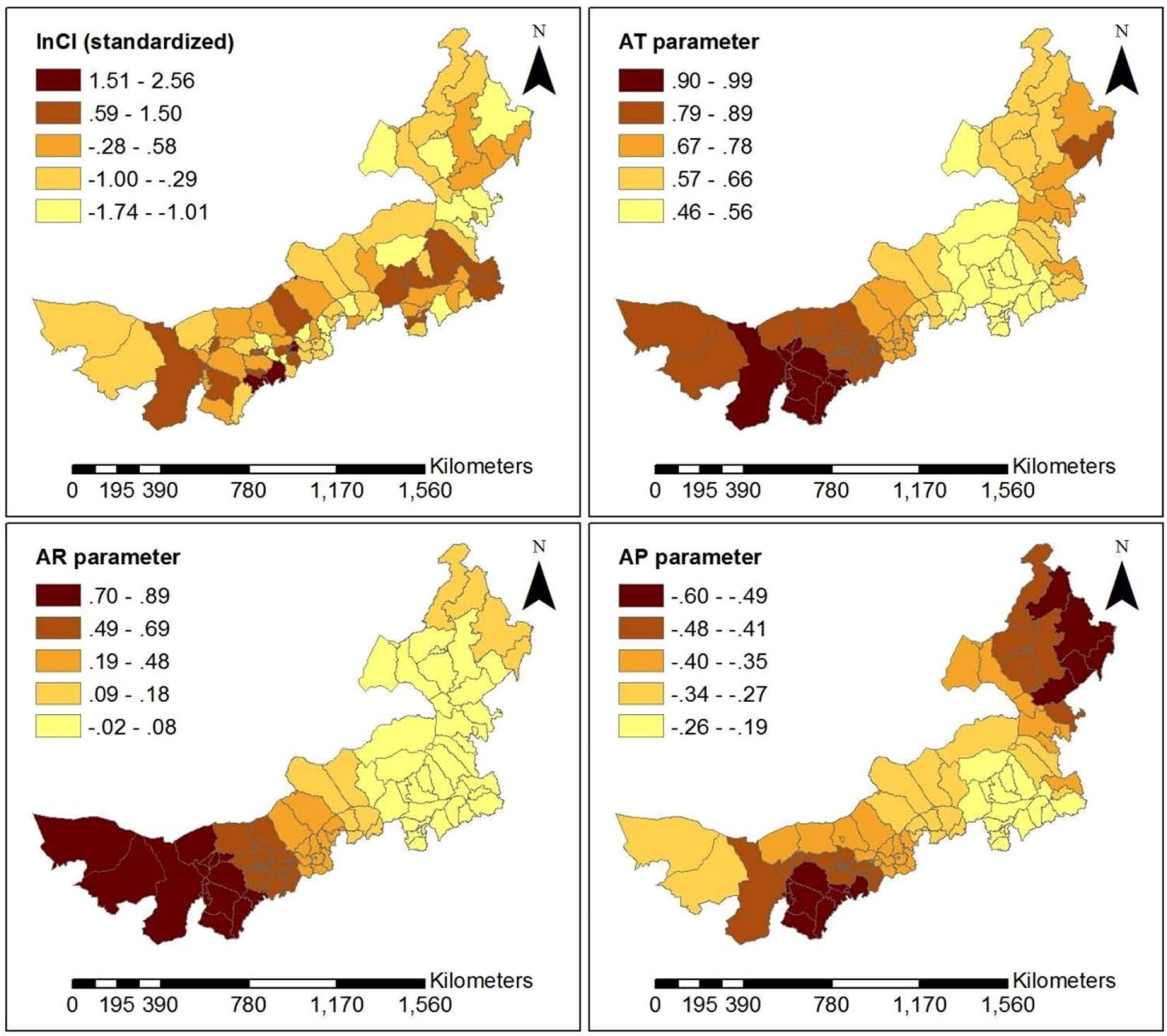
Spatially varying surface of estimated parameters for the GWR model in June, 2016.

**Figure 7:**
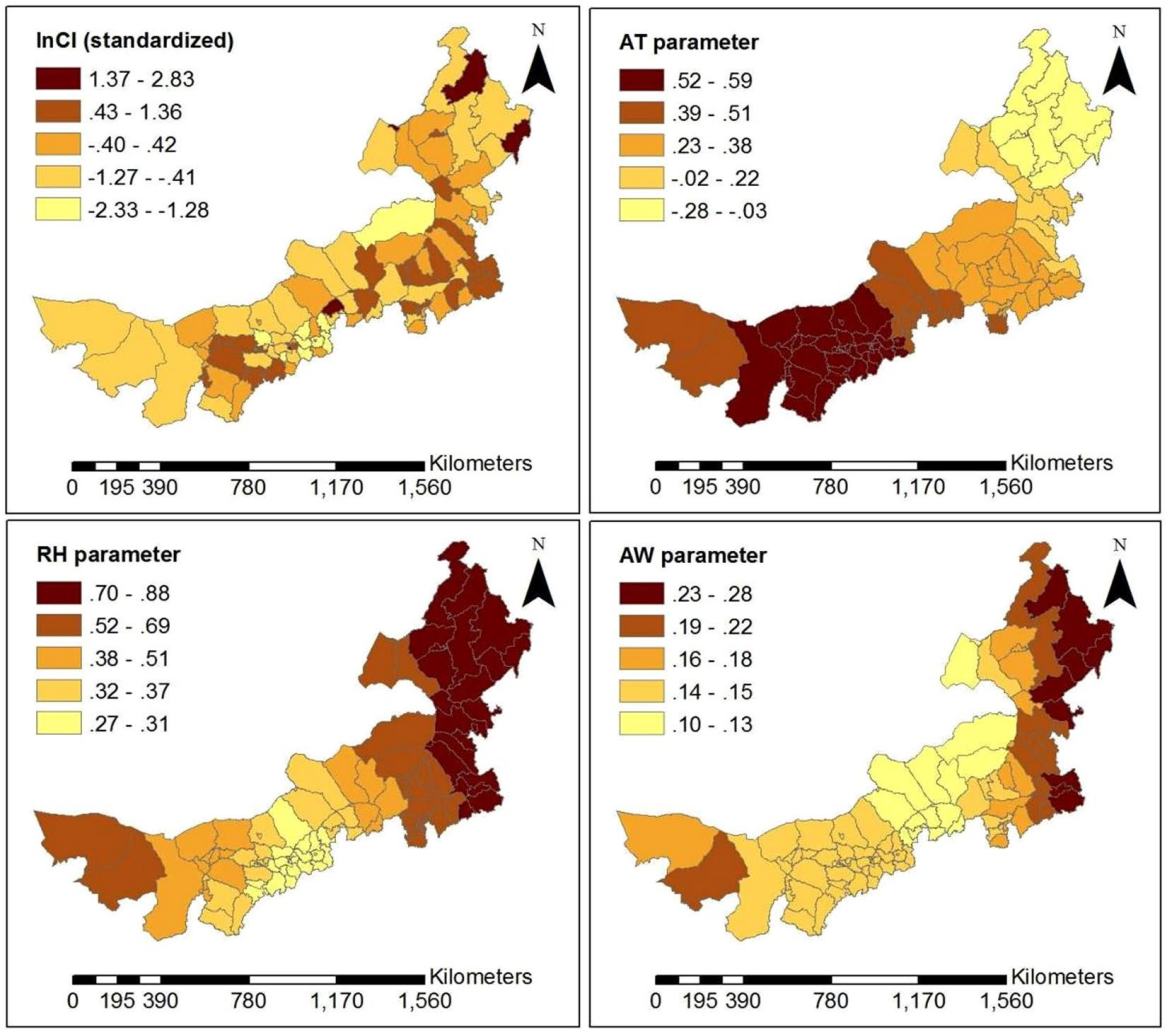
Spatially varying surface of estimated parameters for the GWR model in September, 2016.

**Figure 8:**
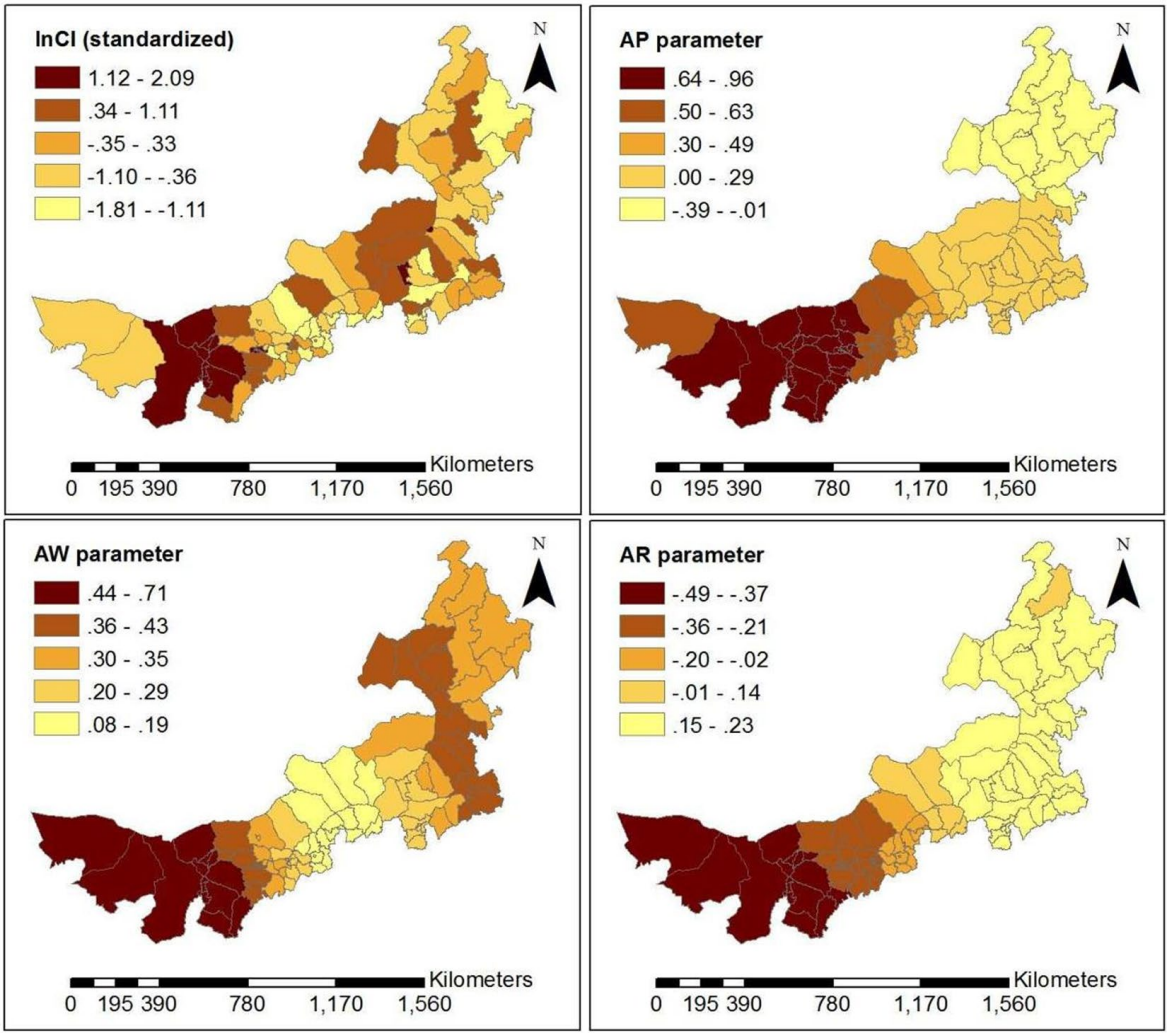
Spatially varying surface of estimated parameters for the GWR model in December, 2016.

The GWR model’s statistics significance and the significance of estimated local parameters were tested by the method presented in literature^25^ for each month from June to December, in 2016. The test results show that the all models were statistically significant, except for models based on data for July and October (Table 2). Among all risk factors (Table 2), AR has the highest pass rate for test on statistical significance and is the most stable, averaging 57.46%. AT is the most statistically significant in summer, with an average of 45.68%. AW is the most statistically significant in winter, with an average of 26.48%, which may be because wind speed is higher than in summer and high wind speed is contribute to the spread of viruses. AP has the higher pass rate for statistical tests in summer and winter, averaging 33.54%. RH is relatively randomly distributed at time period, averaging 20.12%. By the GWR model, each monthly model generated a set of coefficients at county level. These average coefficients of risk factors are shown in Fig. 4. AT and AR have a relatively higher sub-district coefficient than other risk factors during the time period of high disease. Moreover, AT is positively correlated with the incidence of HFMD, while AR has the highest positive correlation with HFMD in summer and is negatively correlated in autumn and winter. RH is positively correlated with HFMD and has greater effect in autumn and winter than in summer. AW is negatively correlated with HFMD in summer and positively correlated in autumn and winter. Child population density (CPD) and Per capita GDP (GDP) are not significantly associated with HFMD occurrence for most time periods. For statistically significant monthly model, the local R square values at county level are shown in Fig. 5. The local R square values vary from 0.143 to 0.804 over space. The spatial distribution of local R square provides evidence of the combined statistical effect of the seven influencing factors on the HFMD cumulative incidence. Figures 6, 7 and 8 display a more detailed analysis of seasonal association between weather factors and childhood HFMD. The estimated coefficients of the risk factors significantly vary over space. The directions of the local relationships are different in different seasons, even for the same risk factor. The risk factors play differently important roles in the incidence and transmission of HFMD in different counties. These results show that there are the spatial heterogeneity of the impact of the risk factors on the HFMD incidence and infection.

**Table 2:** Described statistics for the GWR model results (*p < 0.05, **p < 0.01, ***p < 0.001).

Month	R2	Intercept	AT	RH	AP	AW	AR	CPD	GDP
201606	0.314**	98.0%	100.0%	14.7%	16.7%	13.7%	48.0%	0.0%	28.4%
201607	0.144	—	—	—	—	—	—	——	—
201608	0.189**	0.0%	3.9%	0.0%	57.8%	0.0%	100%	0.0%	0.0%
201609	0.238**	0.0%	57.8%	78.0%	0.0%	6.9%	10.8%	0.0%	0.0%
201610	0.106	—	—	—	—	—	—	—	—
201611	0.412**	33.3%	20.6%	5.9%	27.5%	32.4%	52.0%	40.2%	9.8%
201612	0.497***	18.6%	46.1%	2.0%	65.7%	79.4%	76.5%	21.6%	20.6%

## Discussion

In this study, the GWR model was used to speculate the effect of meteorological factors, child population density and Per capita GDP on HFMD occurrence at different time and county levels in Inner Mongolia, China. Here, we will determine which risk factors play various roles in HFMD epidemic in Inner Mongolia during different seasons. Some previous studies on HFMD have indicated that temperature, relative humidity and precipitation have important impact on the transmission of HFMD^10,26,27^. Our study showed that AT and AR played the greatest role in HFMD occurrence. Furthermore, we found that meteorological factors had different effects on the incidence of HFMD in different seasons. It was reported that children in kindergarten and scattered children are more easily infected with HFMD^23,27–29^. However, in this study, we found that CPD was not significantly related to HFMD from June to October in each county of Inner Mongolia, which may be because precautions and awareness of disease in summer and autumn are higher than that in winter among children. Moreover, outdoor activities are inhibited in winter. It was suspected that the children transmitted the viruses to their younger siblings or neighbours at home^30^. It was argued that the impact of public health measures in prevention and control of HFMD could be strong during the summer months^31^.

As can be seen in Fig. 5, the goodness-of-fit of the GWR model in the west and southwest of Inner Mongolia was superior to other regions in June, November and December. However, in September, there was a superior goodness-of-fit in the northeast of Inner Mongolia. According to the analysis results of each set of parameters of the GWR model, we found that AT and AR had the highest pass rate for statistical tests of significance in summer. AR was the most statistically significant and was positively correlated with HFMD from June to August in almost all counties of Inner Mongolia. Because precipitation in Inner Mongolia occurs almost exclusively from June to August. In December, average pressure (AP) of 65.687% counties and average wind speed (AW) of 79.411% counties had higher statistical significance and were positively correlated with HFMD transmission, which may be because high pressure and high wind speed contribute to the spread of viruses. According to the spatial distribution of estimated local parameters for the GWR model and the risk of contracting HFMD (logarithm of the cumulative incidence) (Figs 6, 7 and 8), weather factors played important roles on the incidence and infection of childhood HFMD in different counties and different time periods. The impact of meteorological factors on the incidence of childhood HFMD depended on the season. AT and AR had better explanatory power for the risk of HFMD incidence in the high risk regions (southwest of Inner Mongolia) in summer (Fig. 6). In early autumn (September), RH played an important role for the high risk regions (northeast of Inner Mongolia), while AT had an impact effect on HFMD occurrence in mid-west and southwest regions (Fig. 7). Because these regions still keep higher temperature in this season compared with other regions of Inner Mongolia. The spatial distributions of the estimated coefficients (Fig. 8) showed that AP and AW had a strong impact on the spread of HFMD, especially for high risk counties in winter, while AR was negatively correlated with the incidence of HFMD in high risk regions.

When the study region is small and homogenous, it is reasonable to assume that the independent variables do not vary significantly over the whole study region, and the relationship between the incidence of disease and potential risks will also not vary. However, the topography and climate change greatly in Inner Mongolia, China. Therefore, it is difficult and unreasonable to keep the spatial homogenous assumption in Inner Mongolia. For the similar conditions, the impact of the potential risk factors on the incidence of childhood HFMD may be more similar in local areas, while for the local areas with great difference of conditions, the impact may be more varying. The GWR model considers adequately spatial heterogeneity of the study data set. By a weighted matrix, the influence of neighbours is also involved in regression model. The present study provides a better explanation and understanding of the spatial patterns and relationships between HFMD occurrence and climate variation in Inner Mongolia. These results of this study may be informative in forecasting the risk of childhood HFMD for different seasons and regions based on climate change.

However, there are also some limitations in this study. First, healthcare resources, such as the number of doctor and hospital bed per capita, may play an important role in HFMD risk. Since we have no available healthcare resources data for the present study, we did not discuss their effect on the HFMD risk. In the further study, healthcare resources’ roles on HFMD risk should be addressed. Second, the present study focused on the spatial analysis of local nonstationary processes. However, beyond space, time is also an essential dimension pertaining to social activities and environmental processes. It has been reported that meteorological factors play important roles in HFMD incidence and epidemic by means of time-series analysis models^32–37^. Further research should be account for local effects in both space and time. Furthermore, the combination effects of two weather factors were not analyzed in the present study. It was reported that the combination of two weather factors enhanced the risk of HFMD incidence, especially for the combination of temperature and precipitation^28,29^. Although there are some limitations, we believe that our findings are beneficial in understanding the seasonal effects of weather factors and the spatial correlation on the incidence of HFMD, which may provide some suggestions to authorities in the development of efficient prevention strategies for different locations in different seasons.

As conclusion, this study analyzed the distribution of the incidence of HFMD in Inner Mongolia from June to December, 2016. The descriptive analysis of data showed that HFMD is serious in some counties of Inner Mongolia and the incidence of HFMD has spatial clustering. Furthermore, the GWR technique was used to explore the effect of potential risk factors on HFMD occurrence in Inner Mongolia. We found that average temperature and average rainfall played important roles on the incidence of HFMD. The effects of AT and AR on HFMD were the most statistically significant in summer, while the effects of AW and AP were statistically significant in winter. RH played an important role in the transmission of HFMD in early autumn. Our results could be helpful in the risk assessment for HFMD epidemic in local regions, and provide instructions for government to rationally allocate public health resources.

## Methods

### Data Collection

In this study, daily reported cases of HFMD in Inner Mongolia from January to December, 2016 were obtained from the Inner Mongolia Center for Disease Control and Prevention. The childhood HFMD cases (aged 0~9 years) were chosen as research population. The data of children population aged 0~9 years and Per capita GDP were obtained from the Inner Mongolia Autonomous Region Bureau of Statistics. Here, the HFMD monthly cumulative incidence (say CI) was calculated by setting monthly HFMD cases (*O*(*i*)) aged 0~9 years as the numerator and the number of the population (*P*(*i*)) aged 0~9 years as denominator. The data of monthly climate factors collected from 119 meteorological stations distributed within 102 counties in Inner Mongolia Autonomous Region, China was obtained from the Ecological and Agricultural Meteorology Center of Inner Mongolia Autonomous Region. Meteorological factors consist of monthly average temperature, humidity, pressure, wind speed and rainfall for each county. Geographic information data of Inner Mongolia Autonomous Region, consisting of the counties’ administrative regions and areas by name and code, was obtained from the Chinese National Administrator of Surveying, Mapping and Geo-Information.

To reduce the spatial variance of CI of HFMD, a Hierarchical Bayesian model was adopted^38^. One important purpose of application of this model is to improve the precision of the estimator of relative risks by ‘borrowing’ strength from other subregions in the study region. In model, *r*(*i*) represents the probability that any child in the *i*^*th*^ county has HFMD, and *O*(*i*)/*P*(*i*) is its maximum likelihood estimate. A model with an unstructured and a structured random effects is to assume the logit transformation of the relative risks logit(*r*(*i*))^38^,

1 $$O(i)\sim B(r(i),P(i));$$

2 $$\,{\rm{logit}}\,(r(i))=\mu +\nu (i)+e(i)$$

where *μ* represents mean effect of the disease risk, *e*(*i*) is independently distributed $N\,(0,{\sigma }_{e}^{2})$, the term $\nu (i)$ is a spatially structured variable and describes the spatial dependence of the relative risk, and is defined by the intrinsic Gaussian auto-regression.

3 $$\nu (i)|\nu (j)\sim N(\sum _{j=1}^{n}\,{w}_{ij}^{\ast }\nu (j),{\sigma }_{\nu }^{2}/\sum _{j=1}^{n}\,{w}_{ij})$$

where *j* denotes the neighbour of site *i*, weights matrix *W*{*w*_*ij*_} is assumed that *w*_*ij*_ = 1 if *i* and *j* are neighbours, 0 otherwise, ${W}^{\ast }\{{w}_{ij}^{\ast }\}$ denotes the row standardized form of weighted matrix *W*. In this model, the flat gamma distribution was used as prior distribution of the hyper-parameters, namely, $1/{\sigma }_{e}^{2}\sim Gamma\,(0.001,0.001)$ and $1/{\sigma }_{\nu }^{2}\sim Gamma\,(0.5,0.0005)$. A non-information prior was taken as the distribution of *μ*. log (*r*(*i*)) was used to denote the logarithm of the expected CI in the *i*^*th*^ county. This Hierarchical Bayesian model was solved by MCMC simulation in WinBUGS1.4, and the length of burn-in sequence is 5500.

### GWR model and parameter estimation

To explore spatial nonstationarity, the traditional regression model is extended to a simple, but effective technique, named geographically weighted regression. This model allows that there are different relationships at different sites across space. Accordingly, the local rather than global parameters can be estimated. So the GWR model can be expressed as

4 $${y}_{i}={\beta }_{0}({u}_{i},{v}_{i})+\sum _{j=1}^{p}\,{\beta }_{j}({u}_{i},{v}_{i}){x}_{ij}+{\varepsilon }_{i},\,i=1,2,\ldots ,n$$

where ${\{{y}_{i};{x}_{i1},{x}_{i2},\ldots ,{x}_{ip}\}}_{i=1}^{n}$ are the observations of the response variable *Y* and the associated variables ${X}_{1},{X}_{2},\ldots ,{X}_{p}$, ${\{{u}_{i},{v}_{i}\}}_{i=1}^{n}$ denote the spatial location coordinates where data is collected, ${\{{\beta }_{j}(u,v)\}}_{j=1}^{p}$ are regression coefficients which are unknown functions of location coordinates, ${\{{\varepsilon }_{i}\}}_{i=1}^{n}$ denote errors which are assumed to be independent and identically distributed random variables with zero mean and the same variance *σ*^2^ > 0. In the GWR model, the coefficients are specified to the functions of location coordinates ${\{{u}_{i},{v}_{i}\}}_{i=1}^{n}$ rather than assumed to be constant. In other words, the GWR model allows the parameters estimates to vary over space, and it is likely that spatial nonstationarity can be captured.

### Testing for goodness of fit and variation of each set of parameters

Utilizing residual sum of squares and its approximation distribution, we can test whether a GWR model describes a given data set significantly better than an OLS model does^25^. And then Leung, Mei, and Zhang^25^ introduced a test statistic to test whether each set of parameters in the model varies significantly over the study space based on the sample variance of the estimated parameters *β*_*j*_(*u*_*i*_, *v*_*i*_) $(i=1,2,\ldots ,n)$ for a given *j* (1 ≤ *j* ≤ *p* + 1).

## References

[1] World Health Organization. A guide to clinical management and public health response for hand, foot and mouth disease (HFMD). *WHO*; Available at: https://www.wpro.who.int/emergingdiseases/documents/HFMDGuidance/en/ (2011).

[2] Zeng H (2015). The Epidemiological Study of Coxsackievirus A6 revealing Hand, Foot and Mouth Disease Epidemic patterns in Guangdong, China. Sci. Rep..

[3] Yang F (2011). Survey of enterovirus infections from hand, foot and mouth disease outbreak in china, 2009. Virol. J..

[4] Chan LG (2000). Deaths of children during an outbreak of hand, foot, and mouth disease in sarawak, malaysia: clinical and pathological characteristics of the disease. For the Outbreak Study Group. Clin. Infect. Dis..

[5] Fujimoto T (2002). Outbreak of central nervous system disease associated with hand, foot, and mouth disease in Japan during the summer of 2000: detection and molecular epidemiology of enterovirus 71. Microbiol. Immunol..

[6] Zhang Y (2009). An outbreak of hand, foot, and mouth disease associated with subgenotype C4 of human enterovirus 71 in Shandong, China. J. Clin. Virol..

[7] Cheng H, Zeng J, Li H, Li Y, Wang W (2015). Neuroimaging of hfmd infected by ev71. Radiology of Infectious Diseases.

[8] Xing W (2014). Hand, foot, and mouth disease in China, 2008–12: An epidemiological study. Lancet. Infect. Dis..

[9] Zhang W, Du Z, Zhang D, Yu S, Hao Y (2016). Boosted regression tree model-based assessment of the impacts of meteorological drivers of hand, foot and mouth disease in Guangdong, China. Sci. Total Environ..

[10] Ma E, Lam T, Wong C, Chuang SK (2010). Is hand, foot and mouth disease associated with meteorological parameters?. Epidemiol. Infect..

[11] Lee CC, Tang JH, Hwang JS, Shigematsu M, Chan TC (2015). Effect of Meteorological and Geographical Factors on the Epidemics of Hand, Foot, and Mouth Disease in Island-Type Territory, East Asia. Biomed Res. Int..

[12] Yu X, Yi W, Niu R, Hu Y (2016). A combination of geographically weighted regression, particle swarm optimization and support vector machine for landslide susceptibility mapping: a case study at wanzhou in the three gorges area, china. Int. J. Environ. Res. Public Health.

[13] Li X, Wang L, Liu S (2016). Geographical analysis of community resilience to seismic hazard in southwest china. Int. J. Disaster Risk. Sci..

[14] Holloway P, Miller JA (2015). Exploring Spatial Scale, Autocorrelation and Nonstationarity of Bird Species Richness Patterns. ISPRS Int. J. Geo-Inf..

[15] Wang Q, Ni J, Tenhunen J (2005). Application of a geographically-weighted regression analysis to estimate net primary production of chinese forest ecosystems. Global Ecol. Biogeography.

[16] Huang Y, Leung Y (2002). Analysing regional industrialisation in jiangsu province using geographically weighted regression. J. Geogr. Syst..

[17] Zhang Y (2009). The epidemiologic and virological analysis of an outbreak of hand, foot, and mouth disease in Inner Mongolia in 2007. Chin. J. Virol..

[18] Li J, Zhao R, Su JM (2013). Epidemiological Investigation of an outbreak of hand, foot and mouth disease caused by CoxA16 virus. Chin. J. School Health.

[19] Hai Y (2015). Epidemic characteristics of hand-foot-mouth disease in inner mongolia, 2008–2013. Chin. J. Public Health.

[20] Tian XL (2013). Epidemiology of hand foot and mouth disease in Inner Mongolia, 2008–2011. Disease Surveillance.

[21] Cheng XS (2017). Epidemiological characteristics and etiological analysis of hand foot and mouth disease in the Inner Mongolia during 2008 to 2015. Chin. J. Viral. Dis..

[22] Chu D (2017). Epidemiology of hand foot and mouth disease in Inner Mongolia, 2011–2015. J. Dis. Monitor and Control.

[23] Hong ZM, Wang XL, Guo WD, Wei LD, Hao H (2017). Temporal-spatial scan clustering analysis on hand-foot-mouth disease during 2009 to 2014 in Inner Mongolia. Mod. Prev. Med..

[24] Hong ZM, Hao H, Wang XL, Wang WR, Wei LD (2017). Analysis of spatio-temporal epidemiology of severe hand, foot and mouth disease in Inner Mongolia from 2009 to 2016. Chin. J. Dis. Control Prev..

[25] Leung Y, Mei CL, Zhang WX (2000). Statistical tests for spatial nonstationarity based on the geographically weighted regression model. Environ Plann A.

[26] Wang J (2011). Hand, foot and mouth disease: Spatiotemporal transmission and climate. Int. J. Health Geogr..

[27] Hu M (2012). Determinants of the incidence of hand, foot and mouth disease in china using geographically weighted regression models. PLoS One.

[28] Dong W (2016). The effects of weather factors on hand, foot and mouth disease in Beijing. Sci. Rep..

[29] Huang Jixia, Wang Jinfeng, Bo Yanchen, Xu Chengdong, Hu Maogui, Huang Dacang (2014). Identification of Health Risks of Hand, Foot and Mouth Disease in China Using the Geographical Detector Technique. International Journal of Environmental Research and Public Health.

[30] Wang Y (2011). Hand, foot and mouth disease in China: Patterns of spread and transmissibility during 2008–2009. Epidemiology (Cambridge, Mass.).

[31] Cao Z, Zeng D, Wang Q, Zheng X, Wang F (2010). An epidemiological analysis of the Beijing 2008 Hand-Foot-Mouth epidemic. Chinese Sci. Bull..

[32] Huang R, Bian G, He T, Lv C, Xu G (2016). Effects of meteorological parameters and pm10 on the incidence of hand, foot, and mouth disease in children in china. Int. J. Environ. Res. Public Health.

[33] Cheng J (2016). Impact of temperature variation between adjacent days on childhood hand, foot and mouth disease during april and july in urban and rural hefei, china. Int. J. Biometeorology.

[34] Yong H (2013). Effect of meteorological variables on the incidence of hand, foot, and mouth disease in children: a time-series analysis in guangzhou, china. Bmc. Infect. Dis..

[35] Huang J (2018). Quantifying the influence of temperature on hand, foot and mouth disease incidence in wuhan, central china. Sci. Rep..

[36] Yang H (2017). Is high relative humidity associated with childhood hand, foot, and mouth disease in rural and urban areas?. Public Health.

[37] Xu J (2015). Impact of temperature variability on childhood hand, foot and mouth disease in huainan, china. Public Health.

[38] Haining R (2003). Spatial Data Analysis: Theory and Practice.

